# Supplementary material for: Infrared Thermography as a Diagnostic Tool for the Assessment of Mastitis in Dairy Ruminants
Source: Animals (Basel). 2024 Sep 16;14(18):2691. doi: 10.3390/ani14182691 (PMC11429297; doi:10.3390/ani14182691)
Supplement: Supplementary file 1 [file animals-14-02691-s001.zip › animals-3179659-supplementary.pdf]

Supplementary material

Table S1. Camera settings and climatic conditions in various thermal imaging studies

Camera	Software - Analysis	Emissivity	Distance (m)	Temperature (ºC)	Humidity (%)	Reference
Flir Inframetrics 760	Freeware ImageJ v.1.28	-	2.0 - 2.5	18.3 (barn)<br>16.3 (outside)	-	[100]
Raytheon	AmberTher software	-	-	19.0	-	[96]
IR Flex Cam Pro	(circle of 40 x 40 pixels above teat)	0.98	0.5	-	-	[74]
IR Flex Cam S	-	-	-	18.0 - 23.0	-	[73]
Fluke Ti 20	-	-	0.5	-	< 72.0	[75]
IR FlexCam S	-	-	0.5	18.0 - 23.0	-	[76]
FLIR System Series-i	QuickReport software	0.97	1.5	9.3	88.3	[91]
FLUKE Ti50FT IR FlexCam	Smart View 3.2 software	-	1.0	15.3 - 16.9	41.0 - 44.0	[98]
IRI 4010	(Rectangle of 55 x 40 pixels at the center of cisternal part)	0.98	0.5	12.2 ± 0.74	-	[92]
FLIR B20 HSV	Thermocam Researcher 2.8 Pro Version Nero (polygons, rectangles, lines)	0.96	1.8	-	-	[77]
FLIR P25	ThermaCam Researcher Basic (ten random spots of each quarter & entire teat end)	-	1.0	14.0 - 22.0	72.0 - 94.0	[78]
ThermaCam P640	FLIR Tools 2.0 Pro	0.98	2.0	-1.0 and 18.0	35.0 and 75.0	[80]
Flir T430sc	Thermovision LabVIEW toolkit 3.3	0.98	0.8	2.6 - 16.2	58.0 - 95.0	[81]

Flir i5	Flir Quick Report 1.2	0.98	1.0	21.4 - 26.6	RH: 62.6 - 92.4	[82]
-	IRBIS 3 standard (circle of 40-pixel diameter above teat)	-	-	-	-	[102]
Thermo GEAR-G120 EX	LabView	0.98	0.6	6.0 - 10.0	-	[83]
Thermo Gear -G120 EX - Nippon Avionics	InfRec analyzer NS9500 Lite - version 2.7A (rectangles of 5x25 pixels at the base, centre, and 1cm above teat tip)	0.98	0.5	15.0 - 25.0	67.0 - 78.0	[97]
ThermaCam B20 HSV	Image recognition software "active shape model", and manually with ThermaCam Researcher Pro 2.8 (polygon tool)	0.96	1.8	-	-	[105]
Flir A615	Deep learning (EFMYOLOv3)	0.98	1.5	-	-	[108]
Fluke TiS10	Smart View 4.3 software - polygon marker	0.98	1.0	32.1	RH: 52.0<br>THI: 75.0 - 80.0	[84]
Flir T360	Flir quick report 1.2 (horizontal/vertical rectangle/lines; spots inside the rectangles; diagonal lines)	0.98	1.5	21.0 - 35.0	52.0 - 95.0	[103]
FLIR T450sc	Flir Tools 5 - Research IR - MatLab (roipoly tool)	-	1.0	16.7	84.2	[85]
Guide C400M	-	0.98	0.3 - 1.0	2.0 - 6.0	-	[106]
Flir A310	Deep learning (YOLOv5)	0.98	1.8 (passage)<br>1.0 ground)	Automatically	Automatically	[107]
Flir E8	Flir professional tool	0.98	0.7	-	-	[95]
Flir I5	Flir Quick Report 2.1	0.98	1.0	Morning: 18.3 - 23.4<br>Evening: 23.7 - 31.3	RH: 60.2 - 97.4<br>THI: 69.8 - 78.7	[101]

Flir 310	Deep learning (YOLOv7, CenterNet	0.98	1.8 (railing)<br>1.0 (ground)	-	-	[109]
DarviDTL007	Darvi TI analysis software	-	1.0	Dry bulb thermometer: 31.5<br>Wet bulb thermometer: 27.0	RH: 73.6<br>THI: 82.7	[86]
Flir E-60	ThermaCam Researcher Pro 2.10 software			-		[99]
Fluke Tix 580	Smart view classic software 4.4 (elliptical marker)	0.95	0.8 (ground)<br>1.0 (animal)	-	76	[87]
DarviDTL007	Deep learning (CNN),<br>Darvi TI analysis software	-	1.0	24.0 - 28.5	RH: 68.7<br>THI: 78.4	[110]
DarviDTL007	Darvi TI analysis software	-	1.0	Dry bulb thermometer: 14.5 - 38.6<br><br>Wet bulb thermometer: 12.3 - 31.0	RH: 51.5 - 92.9<br>THI: 52.3 - 88.8	[90]

RH: relative humidity, **THI**: thermal-humidity index
